# The Safety of Systemic Treatments That Can Be Used for Geriatric Psoriasis Patients: A Review

**DOI:** 10.1155/2012/367475

**Published:** 2012-05-28

**Authors:** Jillian W. Wong, John Y. M. Koo

**Affiliations:** ^1^University of Utah School of Medicine, Salt Lake City, UT 84132, USA; ^2^Psoriasis and Skin Treatment Center, Department of Dermatology, University of California, San Francisco (UCSF), San Francisco, CA 94118, USA

## Abstract

*Background*. Patients with moderate-to-severe psoriasis are often treated with systemic immunosuppressant agents that decrease immune system function. For the elderly, these medications are often problematic due to their already immunosuppressed state and comorbidities. However, there are few studies examining the effects of these medications on the elderly age group. Therefore, there is often discomfort among dermatologists treating elderly patients with psoriasis in utilizing systemic agents, frequently resulting in inadequate treatment. *Objective*. We review the safety profiles of systemic treatments often used to treat psoriasis and their possible adverse risks to the geriatric population. *Methods*. We conducted a search of PubMed's Medline database of articles published from 2000 to 2011, which resulted in 14 articles. *Conclusion*. Treating geriatric patients with moderate-to-severe psoriasis remains a challenge due to immunosenescence and comorbidities. More studies focusing on psoriasis treatment safety in the geriatric population are needed.

## 1. Introduction

 Psoriasis is a chronic, debilitating skin disease that affects approximately 2.6% of the general population [1]. Patients with psoriasis develop erythematous, scaly, and well-demarcated plaques that are often pruritic and can be painful. Due to its chronic nature, psoriasis increasingly affects the geriatric population. A US study in 1991 reported that the highest rate of psoriasis, 113/100,000 population, occurred in the 60- to 69-year age group [2]. With the growing geriatric population expected to compose 25% of the US population by 2020, the prevalence of psoriasis is also expected to rise [3].

There are two key challenges for treating an older patient with psoriasis. The first key challenge involves the already immunosuppressed state of the elderly, also known as immunosenescence. Immunosenescence is a term that describes the immunosuppressed state of the elderly due to natural aging [4]. This is due to a marked decrease in T-cell function with aging as well as other age-related changes in innate immunity [5]. As a result of immunosenescence, the elderly are at a higher risk than younger age groups to develop cancer, infectious disease, neurodegenerative disease, and chronic inflammatory diseases such as atherosclerosis. However, immunosuppressive therapies are considered the mainstay for managing psoriasis and make up the majority of systemic psoriasis treatments. The second key problem in treating geriatric patients with psoriasis is that the elderly often have comorbid illnesses that can be worsened by adverse effects of psoriasis therapies. Older patients frequently have one or more comorbid illnesses that include cardiovascular disease, kidney insufficiency, liver disease, dementia and cognitive disability, diabetes, metabolic syndrome, and obesity, to name a few.

There are few studies examining the effects of these systemic medications on the elderly age group. Therefore, there is often discomfort among dermatologists treating elderly patients with psoriasis. Herein, we review the safety profile of systemic agents often prescribed for psoriasis and their possible adverse effects on the geriatric population.

## 2. Methods

We conducted a search of PubMed's Medline database of articles published from 2000 to 2011 to include only the most recent literature. Articles containing the key words *geriatric population*, *elderly, immunosuppression,* and *psoriasis* were reviewed. In addition, different treatment modalities including *biologic agents, acitretin, cyclosporine,* and *methotrexate *were combined with the key words *geriatric* and* elderly*. The search was limited to articles in English. The search initially resulted in 338 articles. Article abstracts were reviewed for relevance to the subject matter. We examined reference lists to identify any missing articles. Articles included in the review specifically discussed the use of systemic agents in the elderly, the safety profiles of the systemic agents, and/or adverse risks to the elderly. Articles excluded from the review included studies and other review articles that did not specifically discuss the use of systemic agents in the elderly population or the safety profiles of systemic agents.

## 3. Results

After a thorough assessment of articles in the Medline database, 14 relevant articles were included in this review. 4 Food and Drug Administration (FDA) package labels were also incorporated in the review.

Systemic immunosuppressive therapies for psoriasis include biologic agents such as adalimumab, infliximab, etanercept (tumor necrosis factor (TNF) *α* blockers), ustekinumab (interleukin 12/23 monoclonal antibody), and alefacept (T-cell inhibitor). In the treatment of psoriasis, there are also options of nonbiologic immunosuppressive agents that include cyclosporine and methotrexate and a nonimmunosuppressive systemic agent, acitretin.

### 3.1. Systemic Agents and Immunosuppression

Immunosuppression is a concern when using biologic agents. Adalimumab and infliximab have been demonstrated to increase tuberculosis risk [6], histoplasmosis [7], and other granulomatous diseases [8] as well as herpes zoster [9] and malignancy [10]. Ustekinumab has been implicated to increase cardiovascular risk due to earlier phase II research findings, although later data did not demonstrate such risks. In phase II trials of ustekinumab, more adverse cardiovascular events (MACE) such as heart attack, stroke, and sudden cardiac death were found in the experimental group than the placebo group in psoriasis trials [11]. However, this has not been shown in phase III trials or four-year long-term data.

There have been some studies that compare the use of immunosuppressive agents between older and younger cohorts with rheumatologic diseases, such as rheumatoid arthritis, psoriatic arthritis, and ankylosing spondylitis. Safety data extrapolated from the literature of rheumatologic diseases raises concern for using these agents in the elderly. A study of all anti-TNF *α* agents in patients with rheumatoid arthritis showed that there was a significant overall risk of increased serious infections of the elderly over age 65 compared to younger cohorts [12]. The study compared the risk of serious infections in 11,798 anti-TNF treated patients and 3,598 nonbiologic treated patients from the British Society for Rheumatology Biologics Registry [12]. The consequence for this study is that physicians are less likely to prescribe biologic agents to the elderly because of their already decreased immune function. However, one study demonstrated long-term safety of etanercept on elderly patients with rheumatic diseases. The study measured serious adverse events, infectious events, medically important infections, and deaths in 597 geriatric patients over the age of 65 and 3,296 nongeriatric patients with rheumatic diseases [13]. It found no statistically significant difference in safety between elderly and younger age groups [13].

A study by Militello et al. compared the efficacy and tolerability of etanercept for the treatment of psoriasis in elderly and nonelderly populations. The study found that there were no significant differences in the quality of life, as measured by the Dermatology Life Quality Index (DLQI), and in efficacy, as measured by Psoriasis Area and Severity Index (PASI) [14]. Although it showed that there was a significant increase in serious adverse events in the elderly group, these events were reported as not associated with treatment with etanercept. In addition, injection site reaction events were not significantly different between the older and younger age groups [14].

Furthermore, FDA labeling for biologic agents raises concerns for safety in the elderly. According to the FDA package insert for adalimumab (Humira), the frequency of serious infections and malignancies was higher in the elderly than in younger cohorts [15]. The package inserts for infliximab (Remicade), ustekinumab (Stelara), and etanercept (Enbrel), however, reported no overall difference in safety in the geriatric population compared to younger age groups [16–18]. The package inserts for infliximab, ustekinumab, and etanercept also warn that because the risk of infection is higher in the elderly in general, caution should be used when administering these medications in the geriatric population.

### 3.2. Systemic Agents and Comorbidities

One biologic agent, ustekinumab, has been implicated in possible increased risk to cardiovascular health. The immunosuppressive agent, cyclosporine has not been studied in elderly with psoriasis specifically but has the potential to cause damage to several organ systems. Elderly patients often have decreased baseline renal impairment due to aging. Cyclosporine can worsen renal insufficiency and can raise the creatinine level. Age-related decline in renal clearance of cyclosporine has been shown in geriatric kidney transplant recipients [19]. Cyclosporine also can increase blood pressure, which is carefully monitored for the side effect of hypertension at each clinic visit. Cyclosporine cannot be used in patients with abnormal baseline serum creatinine and glomerular filtration rate and is used with caution in the elderly [20].

Methotrexate is another effective nonbiologic immunosuppressive psoriasis treatment that can adversely affect multiple organ systems. A major concern for possible harm of methotrexate is to the hepatic system, as methotrexate has been implicated in hepatic fibrosis and cirrhosis. Elderly patients are at increased risk for hepatotoxicity because of an increased tendency for elevated triglycerides, elevated liver function tests, and obesity that are often found in the aging population and are associated with methotrexate-related hepatic fibrosis [20]. A liver biopsy is required with long-term use of methotrexate, a risk for the elderly that may outweigh the benefits of preventing liver damage. Methotrexate renal clearance is inversely proportional to the patient's age and, therefore, increases renal insufficiency in the elderly, making this agent more problematic [21]. Myelosuppression is another rare side effect occurring in the geriatric population associated with this medication [22]. Patients on methotrexate with comorbid diabetes mellitus are at greater risk for cirrhosis and severe liver fibrosis [23].

One systemic agent for psoriasis that is not an immunosuppressant is acitretin. It is also an effective psoriasis treatment. Though not an immunosuppressant, it also has the potential to cause harm to several organ systems, raising concern for use in the elderly. Acitretin is a second-generation retinoid that may increase cardiovascular risk. It can cause hypertriglyceridemia [20, 23]. In addition, it adversely affects the integumentary system by increasing dryness of the skin and mucosal membranes. This exacerbates xerosis that is already more common in this age group.

Another issue that arises when treating the geriatric population is polypharmacy and consequent drug interactions. Old age is associated with decreased excretory kidney capacity, causing higher risk for adverse drug reactions and interactions especially with multidrug regimens commonly seen in this population. Methotrexate, for example, has a potentially fatal drug interaction with trimethoprim, a common mainstream antibiotic [20].

## 4. Discussion

Based on studies and known risks of systemic medications, physicians and patients both face challenges for determining how to adequately treat psoriasis and avoiding increased harm that may outweigh the benefits of treatment. Further research is needed to develop and make possible medications with decreased risk for cancer, infection, and worsening comorbidities. Extensive studies should be carried out to understand the effects of these systemic agents specifically on the geriatric psoriasis population. In addition to research, the management of elderly patients with psoriasis can be improved by educational programs. Currently, many dermatologists are not comfortable with treating the elderly psoriasis patient with these agents and are inadequately treating them by using topical therapies alone. However, a consensus meeting of geriatric dermatology experts should be organized to develop specific guidelines for dermatologists and other physicians about optional treatments for psoriasis in the elderly.

For now, however, without specific guidelines and adequate research into this topic, a more careful management of the elderly patient with psoriasis is needed. A cautious approach can be taken with a thorough history and physical examination. Included in the history, physicians should document all current and past medications as well as drug allergies and side effects of medications to prevent adverse drug interactions and reactions. A careful history of immunosuppressive use is needed to ensure that the cumulative dose of the agent is not beyond the standard recommended by the FDA to cause toxicity to an organ system. The physical examination should be a thorough head-to-toe examination, with particular emphasis on the cardiovascular, respiratory, and musculoskeletal systems. Vital signs should be taken twice, in particular to ensure that the patient does not have hypertension if the physician is planning to prescribe cyclosporine. Extensive laboratory workup is needed to evaluate for hepatic decline (liver function tests), renal insufficiency (creatinine and possibly glomerular filtration rate), hyperlipidemia, electrolytes, low white blood cell count, low platelets, and low red blood cell count that would be considered contraindications for certain systemic therapies.

When prescribing medications, it is important to start with a small dose and then titrate up to higher doses to a defined therapeutic response. Physicians should make an effort to reduce the number of medications the patient needs to take and should regularly check for possible interactions and adverse effects.

For a healthy, active elderly patient with no or limited comorbidities, etanercept can be used for psoriasis treatment with careful monitoring. Etanercept has been the only systemic biologic agent that has shown to be safe in the elderly, possibly due to its lower immunosuppressive ability compared to other biologic agents. However, a larger sample size of psoriasis-specific elderly patients is needed to further confirm its safety.

If an elderly patient has multiple comorbidities and risk factors that make him/her a poor candidate for an oral or injectable systemic agent, phototherapy and strict compliance to topical therapies are recommended. Ultraviolet B (UVB) and psoralen ultraviolet A (PUVA) phototherapy are noninvasive with minimal side effects limited to mild erythema, burning, and blistering. However, the patient may be challenged by the phototherapy box that requires him or her to be able to stand in the unit without mobile support or guard rails to maintain balance. In addition, some geriatric patients lack transportation or have arthritis or hip impairment that prevent them from being able to move themselves to a dermatology clinic for phototherapy. Drug-induced photosensitivity can also occur if a careful drug history is not taken.

## 5. Conclusion

There is heightened concern about the use of systemic psoriasis medications for the elderly. These medications can be very effective and are considered the mainstay of treatment for moderate-to-sever psoriasis. Virtually all new agents being developed to treat psoriasis are immunosuppressants. However, studies have demonstrated increased risk involved in treating elderly patients with these therapies due to increased comorbidities and immunosenescence. Therefore, many elderly patients are not adequately treated and suffer the physical and psychological effects of psoriasis. These include discomfort and pain from psoriatic plaques as well as depression, anxiety, and stigma from the public because the plaques are often disfiguring. Physicians should remember that the goals of treating psoriasis in the elderly are to achieve clinical control of the skin disease, improve quality of life of the patient, and administer safe and tolerable treatments. It is important for physicians and health care workers serving this patient population to understand the risks associated with systemic agents, initiate and engage in research focusing on the elderly to study these agents and also to investigate new psoriasis therapies, and be willing to treat these patients thoroughly and cautiously so that they are able to obtain adequate treatment to decrease suffering of generalized psoriasis.
